# POMO - Plotting *Omics* analysis results for Multiple Organisms

**DOI:** 10.1186/1471-2164-14-918

**Published:** 2013-12-24

**Authors:** Jake Lin, Richard Kreisberg, Aleksi Kallio, Aimée M Dudley, Matti Nykter, Ilya Shmulevich, Patrick May, Reija Autio

**Affiliations:** 1 Luxembourg Centre for Systems Biomedicine, University of Luxembourg, Luxembourg, Luxembourg; 2 Department of Signal Processing, Tampere University of Technology, Tampere, Finland; 3 Institute for Systems Biology, Seattle, USA; 4 Institute of Biomedical Technology, University of Tampere, Tampere, Finland

**Keywords:** *Omics*, Association, Visualization, Ortholog, Phenolog, Genome-wide, Network, Model organism

## Abstract

**Background:**

Systems biology experiments studying different topics and organisms produce thousands of data values across different types of genomic data. Further, data mining analyses are yielding ranked and heterogeneous results and association networks distributed over the entire genome. The visualization of these results is often difficult and standalone web tools allowing for custom inputs and dynamic filtering are limited.

**Results:**

We have developed POMO (http://pomo.cs.tut.fi), an interactive web-based application to visually explore *omics* data analysis results and associations in circular, network and grid views. The circular graph represents the chromosome lengths as perimeter segments, as a reference outer ring, such as cytoband for human. The inner arcs between nodes represent the uploaded network. Further, multiple annotation rings, for example depiction of gene copy number changes, can be uploaded as text files and represented as bar, histogram or heatmap rings. POMO has built-in references for human, mouse, nematode, fly, yeast, zebrafish, rice, tomato, *Arabidopsis*, and *Escherichia coli.* In addition, POMO provides custom options that allow integrated plotting of unsupported strains or closely related species associations, such as human and mouse orthologs or two yeast wild types, studied together within a single analysis. The web application also supports interactive label and weight filtering. Every iterative filtered result in POMO can be exported as image file and text file for sharing or direct future input.

**Conclusions:**

The POMO web application is a unique tool for *omics* data analysis, which can be used to visualize and filter the genome-wide networks in the context of chromosomal locations as well as multiple network layouts. With the several illustration and filtering options the tool supports the analysis and visualization of any heterogeneous *omics* data analysis association results for many organisms. POMO is freely available and does not require any installation or registration.

## Background

Modern high-throughput technologies measuring different *omics* types are constantly producing masses of new data [1-3]. Simultaneously, the various analysis algorithms and association analyses methods applied to these measurements are providing many different types of results [2-6]. Thus, the integration of the data and subsequent visualization of these results are becoming increasingly important and challenging [7].

The different types of analysis algorithms are resulting in various types of associations within the data. Often these methods include correlation-based or integrative data mining algorithms [6], and the results can include genomic feature to genomic feature associations across multiple data types, such as gene expression and chromosome rearrangements. The features can, for example, be genes or genomic positions such as regulatory regions, or they can be also clinical or sample annotations resulting for example from differential expression analysis [3,8]. While the different values or types of data are related with each other, it also becomes necessary and challenging to be able to visualize different types of data and the results of their analysis [7,9,10]. Generally, the results of various analyses are given as text lists and visual illustrations are confounded by different formats, software platforms, and dependencies. However, because most of the genomic data can be organized by its genomic location, it is straightforward and advantageous to utilize the genomic position as a parameter in visualization. Since the majority of resulted *omics* associations can be linked to the physical chromosome positions, genome-wide illustrations can provide new insights to the investigator [9].

Traditional genome browsers such as Integrative Genomic Viewer [11], UCSC Genomic Browser [12] and GBrowse [13] are very useful for viewing biological data with multi-scaled linear tracks but they are not ideal to view gene networks. Cytoscape [14] fills this need and is adept at displaying network interactions and has released CytoscapeWeb [15] and Cytoscape.js beta libraries designed for web programming integration. Given that structural rearrangement events are likely more informative in the context of ordered chromosome circular layout context, there are a limited number of software tools available for circular illustration of the genomic association data, of which Circos [16] is most often used. Circos provides command line options to plot various types of data together into assorted attractive but static circular plots. Circos software requires local installation along with several mandatory Perl core and third party modules. The recent introduction of RCircos [17] successfully draws Circos images with R but implies that its usage is limited to experienced R programmers. DNAPlotter [18] plots interactive user-defined circular and linear genomic tracks. This standalone tool, improved from other published genomic viz tools such as CGView [19], GenomeDiagram [20], GenomePlot [21], GenoMap [22] and Microbial Genome Viewer [23] by combining Jemboss [24] and Artemis [25], flexibly accepts custom text files and relational databases, and the plotted tracks can be filtered and exported. DNAPlotter requires installation and does not support associations. Galaxy [26], web-based and very comprehensive for biomedical analysis and sharing, recently introduced Circster [27] a web-based Circos like visualization as part of its comprehensive pipeline. While Galaxy is available both publically and as a local install, Galaxy visualization functions are only available downstream of its workflows and thus limited to its ecosystem. As such, visualizing *omics* data with such a program requires a certain level of computational experience and multiple programs to illustrate, share and filter the data analysis results. In contrast, the UCSC Interaction Browser [28] and WikiPathways [29] both allow for web visualization and organization of network interactions, but they do not have genomic chromosomal context association views and they lack support for several important model organism references. In addition, as *omics* data includes often thousands of feature values, and there are at total thousands to millions resulted associations, it is vital to support filtering options for exploration and detection of sub-networks from dense and cluttered networks.

To address these issues, we have developed POMO, Plotting *Omics* analysis results for Multiple Organisms. POMO is a free web-based software suite that permits the illustration of associations inferred from *omics* data as filterable circular genome-wide, Cytoscape Web and grid views. Aiming to parallel the diversity of systems biology research, POMO software has built in reference support for human [30] and the following model organisms: mouse [31], zebrafish [32], worm [33], fly [34], rice [35], tomato [36], *Arabidopsis*[37]*, S. cerevisiae*[38] and *E. coli*[39] (See Table 1 for resources). In addition, the program accepts parameters for integration and plotting of genomic homologies and orthologous features of multiple strains of the same organism or closely related species. Multiple text file formats are supported, and associations can be directly uploaded or referenced as URL addresses using modern web browsers. POMO supports the plotting of an unlimited number of rings to highlight genomic annotations and regions of interest, and all results remain private and can be exported and shared as SVG image or TSV text files. The web based (http://pomo.cs.tut.fi) program is a freely available user-friendly tool for genome-wide biological research that does not require any installation or registration. With the wide selection of data visualization options, POMO is a unique tool for all the researchers working with *omics* data analysis, which can be used, for example, to visualize and filter the genomic networks in the context of chromosomal locations as well as multiple network layouts.

**Table 1:** Supported organism references

**Organism**	**Species/build**	**Source**	**URL**
Human	H. Sapiens (GRCh37.p11)	ENSEMBL	http://www.ensembl.org/Homo_sapiens/Info
Fly	D. melanogaster (BDGP5)	Fly base	http://flybase.org/
Mouse	M. musculus (GRCm38.p1)	MGI	http://www.informatics.jax.org/
Worm	C. elegans (WBcel235)	Worm base	http://wormbase.org/
Yeast	S. cerevisiae (EF4)	SGD	http://www.yeastgenome.org/
Zebra fish	D. rerio (Zv9)	ZFIN	http://www.zfin.org/
Arabidopsis	A. thaliana (TAIR10)	TAIR	http://www.arabidopsis.org/
Rice	O. sativa (MSU6)	MSU	http://rice.plantbiology.msu.edu/
Tomato	S. lycopersicum (SL2.40)	SolGenomics	http://solgenomics.net/
E. Coli	K-12 (MG1655)	Ecocyc	http://ecocyc.org/

## Implementation

It is widely accepted that visual networks are valuable for detecting and exploring patterns in large datasets. Genomic network visualizations with multiple perspectives, particularly within chromosomal context can offer insights of key proximal nodes and possible sub-networks. Data mining algorithms produce genome-wide association sets where individual associations are described with either a numerical ranking or weight. The option to filter and iteratively visualize these large data sets is of key importance in exploring and understanding the genomic associations. Our web application addresses and extends these requirements by combining different data types and including the reference genomes of multiple organisms by utilizing modern web programming technologies and components. POMO allows immediate visualization of genome-wide associations and annotations directly from text files while offering grid, Cytoscape and genomic circular context views. Within the genomic circular context, chromosomes are drawn as segments of the circumference; its length is normalized dependent on the nucleotide base length of the displayed organism. *Omics* nodes, which can be labelled as gene names or ids or explicit genomic positions, will be oriented/mapped to these segments, and the associations are represented as an edge between two genomic locations or genes. For additional visual differentiation, the notations are color encoded for different *omics* data types, such as gene expression, copy number variations, or proteomics data. Multiple annotation rings, with support for bar, histogram and heatmap graphs, can also be appended. Outer glyphs are used for representation of genomic features to unmapped nodes, which have no genomic location, such as phenotypic traits or disease state features.

Many labs studying data originating from *omics* studies of different organisms are lacking the personnel and expertise to write customized software for visualizing genome-wide associations. The inclusion of multiple organisms into POMO addresses this need by enhancing the utility and usability of visualization software. POMO supports the newest genome builds of the following organisms: human, mouse, nematode, fly, yeast, zebrafish, *Arabidopsis*, rice, tomato and *E. coli* (Table 1).

**Figure 1:**
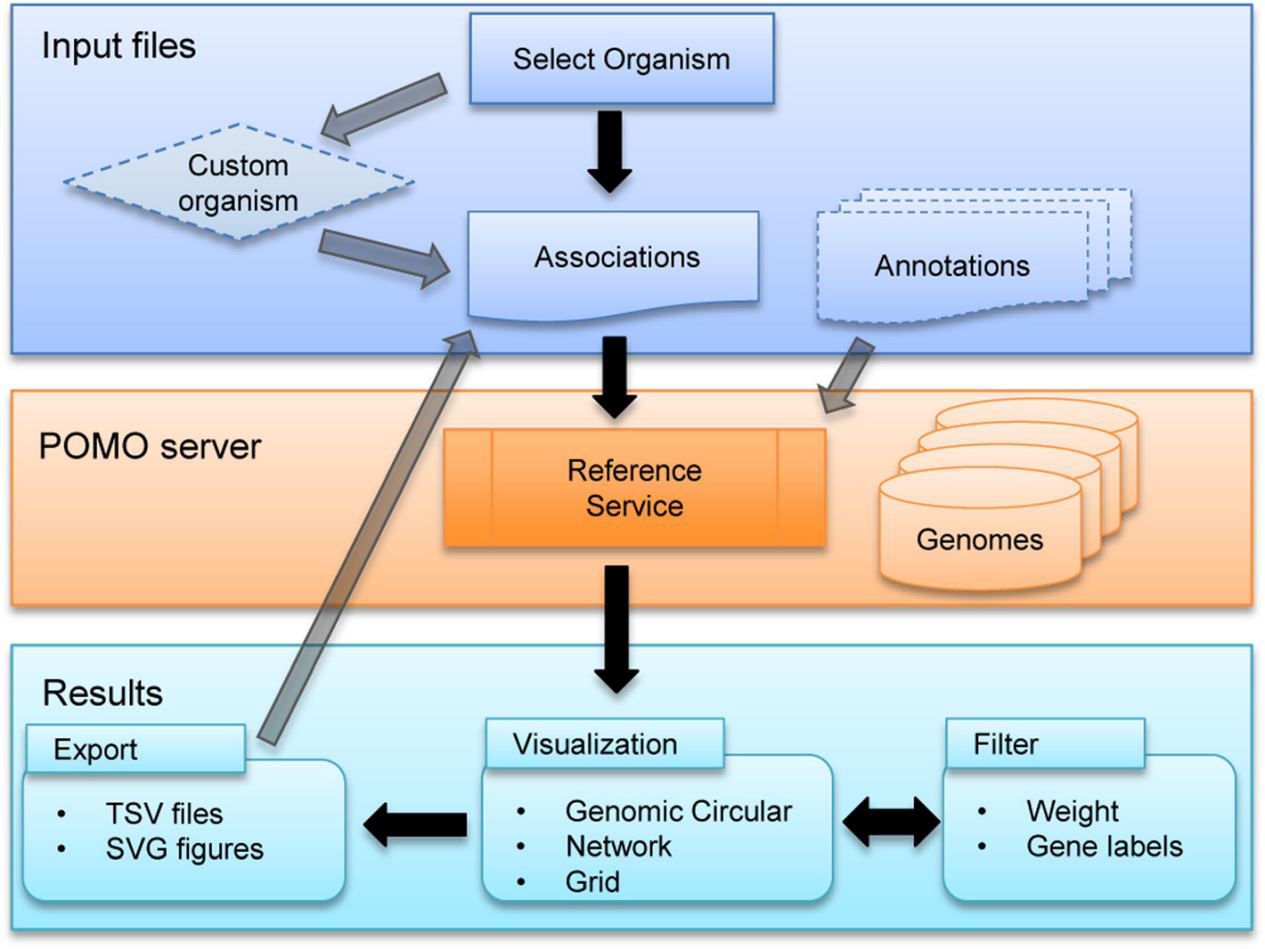
**POMO flow chart.** When using POMO, first the user selects the organism he wants to study. It is also possible to create a custom organism. Second, user uploads the associations and possible annotations as data inputs into POMO. Currently, POMO is supporting 10 different genomes. POMO visualizes the input associations instantly providing multiple options for the views. The weights and labels, including gene sets, such as pathways, can be applied for filtering. All views are exportable as figures and also as text formats which can be directly used as future inputs.

Additionally, POMO provides an interface for a custom/new organism selection. This option allows users to define a new organism, which can be for example an existing organism that POMO does not yet support, parts of an existing organism (chromosomes or contigs), or combination of several species. As outlined in Figure 1, unsupported or custom references can be defined and their associations plotted and exported. In addition, POMO enables pairwise between-organism comparison allowing visualization of in-between associations of genes or genomic locations between different organisms, such as human-mouse or yeast-yeast. The resultant views can be exported as an SVG and converted to publication resolution quality images using free tools like Inkscape. This function will assist labs with communicating and sharing their association findings. The exported filtered text associations can be used as immediate POMO inputs as well. Further, POMO supports direct URL referencing of associations, such as cloud-based files stored on GoogleDrive or DropBox, and thus researchers can communicate their insights visually with fellow collaborators. POMO does not store any upload data thus preserving and addressing security and privacy.

POMO is designed for illustrating *omics* associations directly from text files in circular genomic, network and tabular contexts with dynamic built in organism reference and annotation support. Following graph syntax from math, an edge is defined as two nodes having a link or association. In POMO, this edge can be ranked with a numeric weight, such as a p-value or correlation, or the user can directly mark this association with a color. Input associations can be derived from any data mining method as long as node labels are either gene names, identifiers such as ENSEMBL and ENTREZ or chromosome based positions. This flexibility allows for network nodes to be in non-coding DNA range which leads to complete inclusivity. Non-gene coding events such as promoter sites, copy number variation and other aberrations can easily be integrated and visualized. The program supports mixing gene and non-gene position based node labels. POMO node labels can be either ENSEMBL/ENTREZ id or gene label or position based. Position based nodes are labelled in the form chr:start:end. The nodes may be enhanced with a source type, such as genotype (GENO), gene expression (GEXP) or proteomics (PROT) data. These optional node annotations are encoded to a set of colors that lead to richer and differentiable graphical details. In addition, POMO supports multiple genome wide annotation rings, where the rings are defined in a text file and then uploaded. The syntax allows for pairing of values or colors to a gene or a segment in the chromosome. Syntax details and examples are provided in the Additional file 1. As exhibited in Additional file 1: Figure S10, annotation rings can be represented as bars, histograms and heat maps. Unmapped (PHENO) phenotype associations are visually portrait as outer glyph ticks, where the position represents the genomic position linked to the unmapped feature.

POMO inputs are text files containing genomic results such as interactions or associations. Each edge defines two nodes and the nodes are labelled with a gene name or ENSEMBLE or ENTREZ identifiers. The user can mix the node labels freely and Additional file 1: Table S1 provides more details and examples. Edges can optionally be rank with weights and also directly marked up with an HTML supported color. The supported delimiters along with the file type extensions are spaces (.txt), tabs (.tsv) and commas (.csv). Simple Interaction Format (.sif), which allows for multiple associations to be placed on one line, is also supported. We have also extended the sif format to allow an optional weight or color column.

Utilizing HTML5 FileReader API and modern web browsers, the tool allows uploading of association and annotation text files and then upon chromosome position translation immediately plots the resultant graph. Publically accessible cloud hosted *omics* association files can be read by POMO as an URL parameter. For testing and efficient plotting of small networks, one can declare association edges directly inside the URL parameter. Details and syntaxes are provided in the user guide, Additional file 1. The software includes comprehensive dialogs and messages to report if certain association node labels cannot be mapped to the selected reference. Association weight filtering can be accomplished if numeric values are provided. Moreover, POMO also allows for label set filtering, meaning, *e.g.,* that a list of gene labels, such as members of a particular pathway, can be used to find subsets of the graph. The circular, grid and network views are automatically refreshed on each filtered submit and their iterated graph images can be exported as SVG image file, suitable for publishing or posters with its high definition presentation.

The interface dialog windows are programmed with custom listeners and AJAX events for seamless dynamic document updates. Since JavaScript allows for functions as parameters, these dynamic functions are then being utilized on callback functions upon user selection of organism and file format selection and upload. Object instantiations are also linked to different user interface selections, such as organism determines the genome browser a node click resolution. This Web 2.0 application includes extensive usage of ExtJS layout and panels, and jQuery AJAX with JSON Objects for data exchange. POMO circular visualization is built on top of VisQuick that utilizes Protovis [40] while the network view integrates CytoscapeWeb with custom discrete mappers and data population functions. Built on modern web software principles that include integration of python libraries and SQLite databases, the application can be deployed to all major web servers independent of platform and operating system.

## Results and discussion

Big data is a large and routine part of modern day genomics research; along with troves of public databases, labs are generating different types of genome-wide data from new experiments and various instruments. Various sets of associations, often heterogeneous, are being extracted and by using POMO the investigators can gain insights from the different visual perspectives and layouts. Of particular interest is the genomic circular layout, where nodes are spatially mapped to chromosome arcs on the circumference and the associations are represented as edges between the genome-anchored nodes. Proximal and high degree nodes are revealed instantly, as well as sparse disjoint associations. With usage of filtering by association weight with multiple operators, gene label, or list of gene labels that can be for example pathways, investigators can intuitively find insights from previous uninformative dense networks. It is well known that genome wide visualizations, particularly in circular context, can have limited spatial capacities and dense graphs are not informative. To address this, POMO allows for filtering and edge bundling functions. The edge bundling allows for a node range window and groups the edges if the start and end nodes are within this window. Optionally, a score threshold can be set to exclude valued edges from the bundling (See Additional file 1 for more usage details).

**Figure 2:**
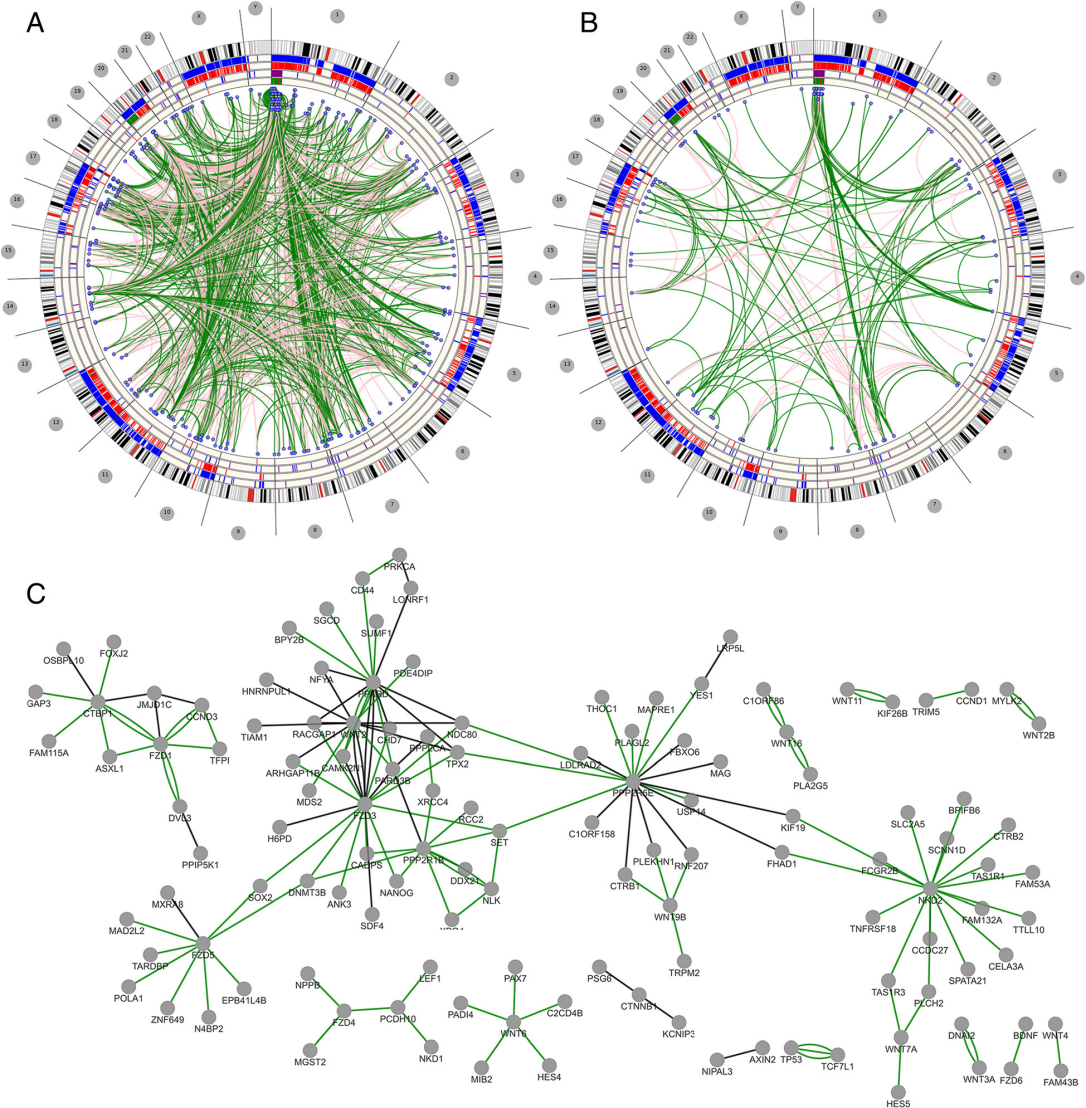
**Illustration of copy number alterations and gene expression value associations in hESC and hiPSC samples.** The figures depict associations detected in human embryonic (hESC) and induced pluripotent stem cell (hiPSC) data and associations with colored based on the correlation value. The file has 45,791 edges and the upload and plotting took 5 seconds. **A)** POMO illustration of the best 2000 correlations shows 2000 edges and with heatmap rings hiPSC/hESC expressing high/low gene expression with CNV gain and loss. Green edges indicate positive correlations while pink indicates negative. **B)** The result is further filtered using set membership check on the gene list taken from KEGG WNT pathway and the edge weight abs (correlation) > = 0.92, yielding 160 edges. **C)** The result in sub-Figure B is illustrated with the Cytoscape Web radial layout.

POMO can serve as a tool for genome-wide network visual exploration and communicative collaboration since the filtered results can be shared as exported files, images or directly as an URL. Clicking on nodes will open specific Genome Browsers on the selected region window of the specific organism. In the following scenario, POMO is used for integrating and visualizing copy number gains and losses in relation to correlation associations in application of human embryonic stem cells (hESC) and human induced pluripotent stem cells (hiPSC) samples [41,42] (Figure 2). The rings in POMO plot are illustrating the copy number variations together with genes whose expression values have been identified to be associating with the copy number variation. In Figure 2, after the outermost cytoband, the first ring is indicating the areas whose copy number has been altered in hESC samples, while the next ring illustrates the genes whose high expression is associated with gain in copy number (red) and whose low expression is associated with loss in copy number (green) of the same samples [41]. Similarly the fourth ring illustrates the copy number alterations in hiPSC samples [42] and lastly the associated genes with them in the same samples (unpublished observations, Laurila *et al*. submitted). The edges demonstrate correlations between the detected genes computed through all the expression data. Based on the genome-wide figure it is easy to see how there are several genes with copy number alteration in both hiPSC and hESC samples in the chromosome 1, that are highly correlating with other altered genes and are also a part of WNT pathway.

**Figure 3:**
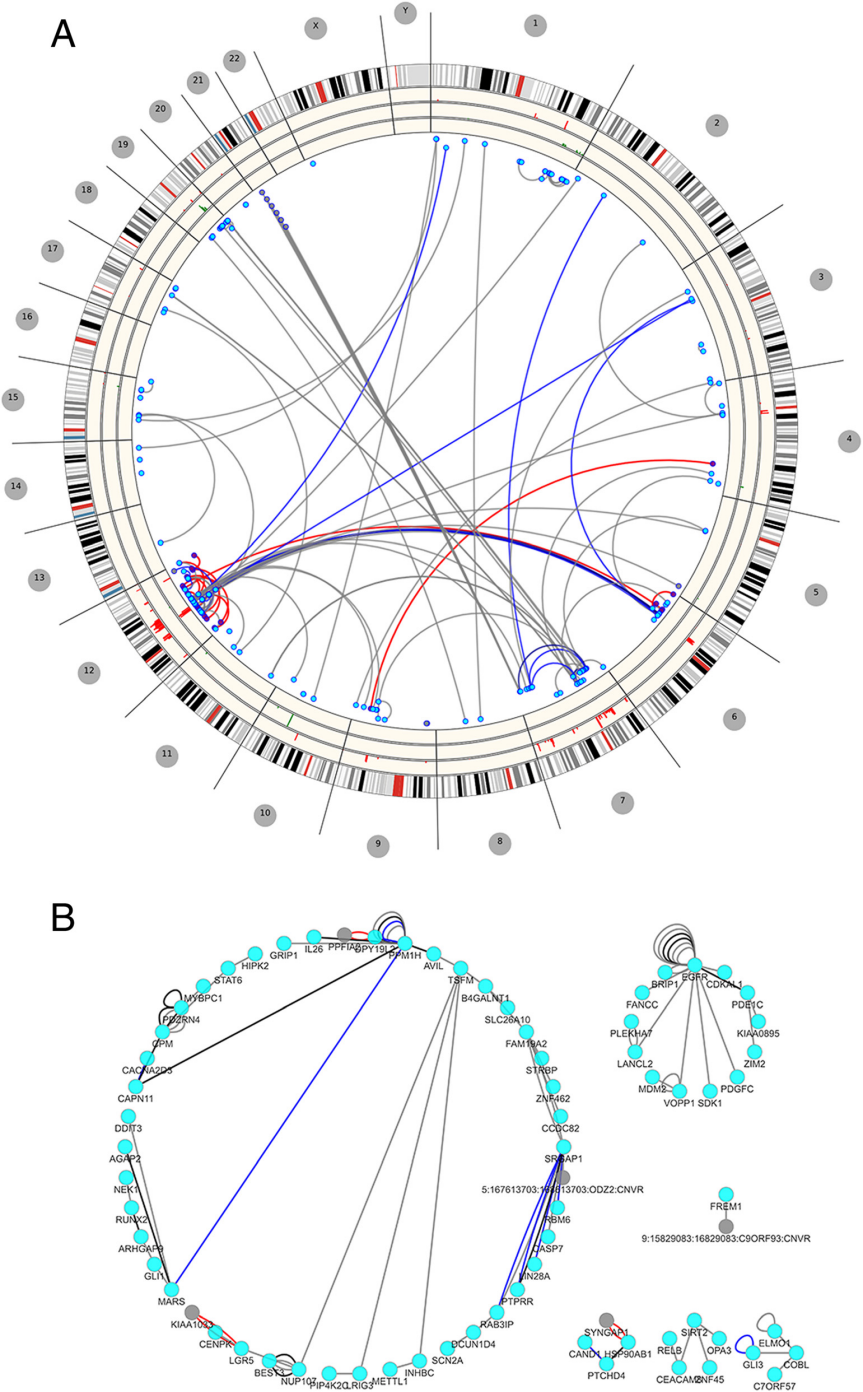
**Plotting genomic structural variations.** The figures depict TCGA GBM [43,44] rearrangements and chromothripsis findings from whole genome sequencing. **A)** Edge colors are used to describe number of supporting reads, with gray < 50 and blue greater than 100. Histogram rings are depicting copy number gain and loss ratios while the inner most ring accounts for possible gene fusion events. **B)** The result is showing the network view of the same data where the single edge associations are filtered.

Genome-wide contexts can be particularly helpful in viewing chromosomal arrangements. Figure 3 depicts TCGA glioblastoma multiforme (GBM) [43] rearrangement and chromothripsis events associated with poor survival [44]. Using data in the accompanied supplement, chromothripsis results are represented as red edges while blue edges demonstrate rearrangements with supporting reads of greater than 100, where grey represents supporting reads of lower than 50. Chromosome region 12q14-15 is considered as a breakpoint-enriched region where oncogenes *CDK4* and *MDM2* are noted to amplify frequently [44]. The inner red ring of the figure demonstrates these elevated amplifications where the next two inner rings represent gains (green) and then genes with evidence involved in fusions.

Another case study is the visualization of high quality yeast protein-protein interactions labelled with ENSEMBL gene ids [45,46]. Released as part of Cytoscape, the file contains 6888 edges and can be directly uploaded into POMO without any data manipulation. A full workflow, including file upload and resolution of chromosome positions using POMO’s reference translator service took 1.9 seconds and then 1 second to plot the default but configurable limit of the first 2000 edges [See Additional file 1: Figure S13]. This is consistent with our randomized testing of 1000 edge sets where the genomic translation service performs around 500 milliseconds and then almost instantaneous plotting. See Table 2 for more details on browser/OS comparisons. Though web based software has a dependence on network connectivity, we have successfully tested the service from different locations. For clarity, plot limits can be set easily with a pull down list and filtering, whether it is label set or scoring based, is always applied on the full association set. The actual plotting relies on browser/client memory. Furthermore, the export of filtered associations can serve as inputs on future POMO sessions. The different views are all updated dynamically and synced with the latest uploaded and filtered results. Users can toggle between the tree, circle, radial and force-directed layouts in the Cystocape Web view.

**Table 2:** Performance benchmarking on yeast protein-protein associations

	**Process**	**Firefox**	**Chrome**
**Windows 7 4 GB RAM 2.6 GHz**	Upload/server translation	1.5 seconds	1.5 seconds
	Browser plotting	1 second	1 second
**Mac OS 10.8 8 GB RAM 1.8 GHz**	Upload/server translation	1.5 seconds	1 second
	Browser plotting	1 second	0.5 second

The network consists of 3025 nodes and 6888 edges. The times given here include uploading/translation (genomic identifiers to chromosome positions) and plotting. Generally, Chrome is faster at plotting while uploads will depend on network speed and geographical location. These tests were done in Finland and Germany on wireless connections. We recommend using relative recent releases of Firefox and Chrome because of HTML5 file uploading and JavaScript libraries dependencies.

**Figure 4:**
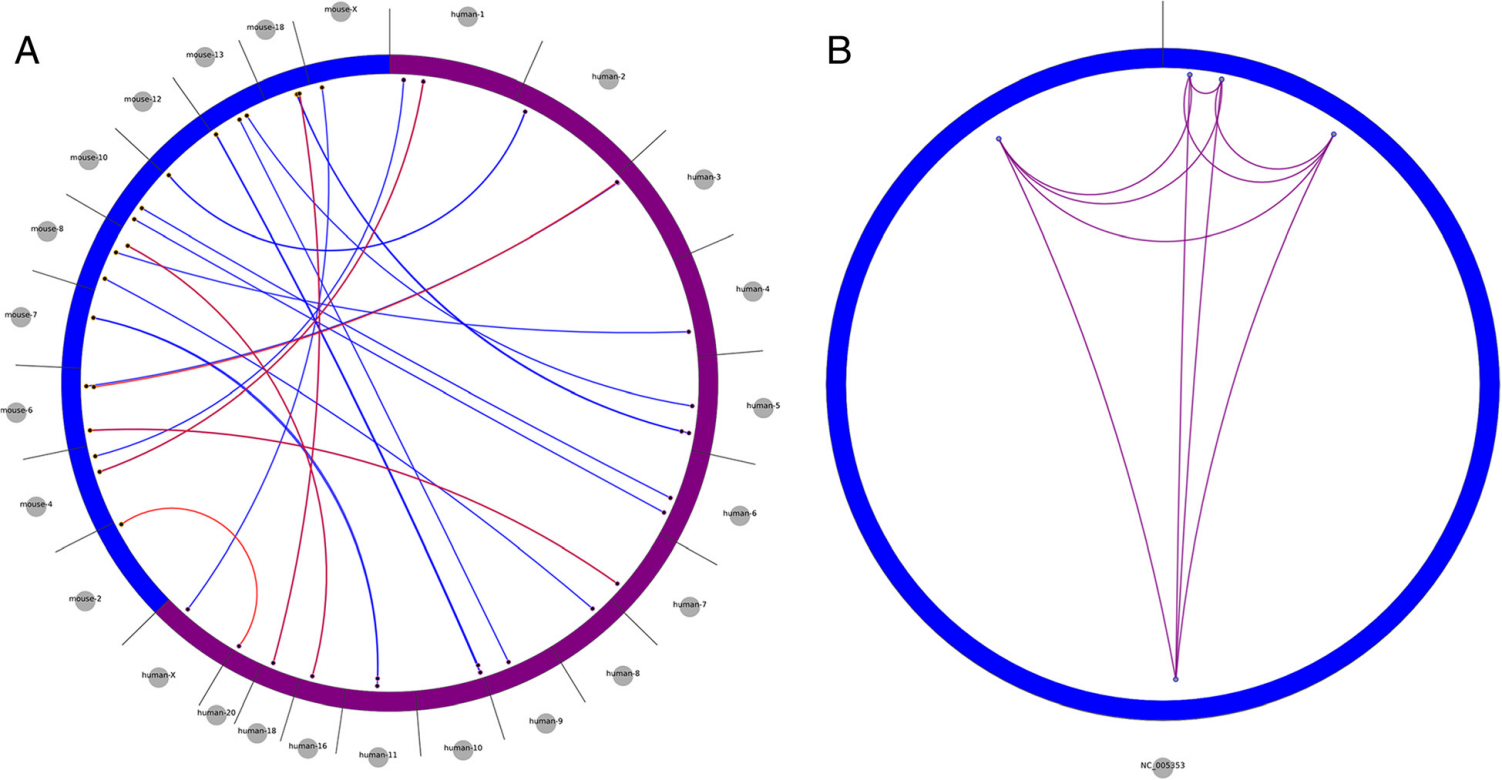
**Mouse-human phenolog homology and custom alga network views. A)** Genome-wide visualization of mouse-human obesity ortholog associations. Blue perimeter stands for mouse while purple is for human, blue edges stand predicted mouse orthologs based on shared human phenotype while red edges indicate ortholog groups shared by human and mouse phenotypes. **B)** Figure shows the chloroplast genome of *Chlamydomonas reinhardtii* (NC_005353) highlighting the chloroplastic part of the *cyt b6f* complex where its nodes and edges consist of the genes *petA, petD, petB, petG, and petL*.

POMO also allows the user to visualize genomic associations between two related organisms, or two distinct strains within the same POMO supported organism. Figure 4A exhibits phenolog [47] orthologs of obesity-abnormal food intake between human and mouse. Edge colors are used to differentiate predicted orthologs and shared orthologs based on observed phenotypes. Using the same interface and selecting custom organism, the user selects the organisms to contrast, and then the input file association node labels are resolved based on the selected reference. Following this workflow, an unsupported organism can be defined by indicating its chromosomes and base lengths. Figure 4B demonstrates the custom function to illustrate the chloroplast genome of the green alga *Chlamydomonas reinhardtii* (NC_005353) [48], highlighting the associations of genes in the *cyt b6f* complex**,** which mediates electron transfer between photosystems (PS) II and I, cyclic electron flow around PSI, and state transitions [49]. More information concerning custom organism options is described in detail in the Additional file 1.

## Conclusions

POMO, freely available for non-commercial research, was designed for life science researchers to easily plot, filter and share genome-wide *omics* data and associations using an intuitive web interface. In supporting different labs studying different organisms, a comprehensive set of model organism genome references are fully integrated to allow for flexible association notations. The unique property, only available in POMO, is allowing the user to illustrate various organisms or closely related organisms together within single view. POMO also includes a detailed user guide, and several example associations and annotations are provided. In future, we will add support for other further organisms and appreciative of user feedbacks to improve the views and interface. For maximal visual impact, different visualization views and network layouts are supported and can be seamlessly toggled with simple clicks. Upon filtering, each view is dynamically filtered and text exports can serve as future inputs while the SVG image export can be converted to publishing quality presentations. POMO is an open sourced project and the code, builds and documentations are available at http://pomo.googlecode.com. In sum, as genome-wide visualizations, particularly interactive and web based, can help researchers to confirm theories and formulate new research questions, POMO can significantly facilitate researchers in finding new biological discoveries among their *omics* data.

## Availability and requirements

**Project name:** POMO: Plotting *Omics* analysis results for Multiple Organisms

**Project home page:**http://pomo.cs.tut.fi

**Operating system(s):** Platform independent

**Programming language:** Python 2.6+, JavaScript, HTML5, SQLite 3.7+

**License:** POMO is available free of charge to academic and non-profit institutions.

**Any restrictions to use by non-academics:** Please contact authors for commercial use.

## Competing interests

The authors declare that they have no competing interests.

## Authors’ contributions

JL, PM and RA conceptualized and initiate the project. JL, RK, PM and RA designed POMO that JL and AK implemented. RK designed and implemented VisQuick. JL designed, implemented and populated the reference databases and translation service stack. JL, RA, and PM drafted the paper. AD, MN and IS contributed important ideas and advices. RA, PM and IS supervised the project. All authors read and approved the final manuscript.

## Supplementary Material

Additional file 1 POMO User Guide. Click here for file
