# Supplementary material for: POMO - Plotting Omics analysis results for Multiple Organisms
Source: BMC Genomics. 2013 Dec 24;14:918. doi: 10.1186/1471-2164-14-918 (PMC3880012; doi:10.1186/1471-2164-14-918)
Supplement: Additional file 1 — POMO User Guide. [file 1471-2164-14-918-S1.docx]

POMO User Guide

Contacts: jake.lin@uni.lu

Code Source and other information: <http://code.google.com/p/pomo/>

Web address: <http://pomo.cs.tut.fi>

Updated Dec 2^nd^, 2013

Content:

1. General purpose
2. Browser Recommendations
3. Interface, Views and Layout
4. Data Upload and Export
5. Filtering
6. Custom/Homology
7. Architecture and Libraries

**1. General Purpose**

Plotting Omics analysis results for Multiple Organisms is an open sourced web application that draws secured association graphs in genomic and network views on user uploaded association and annotation text files. The association network can be further filtered using gene label or label sets; as well as equality operations on provided association weight. The online tool has integrated reference support for human, mouse, zebrafish, fly, worm, yeast, rice, tomato, *Arabidopsis*, and *E. coli*. (See Table S1 for edge syntax and Table S2 for organism sources) Association node labels can be combinations of genomic position based, or ENSEMBL or ENTREZ Ids as well as gene names. Along with the supported organisms, a custom interface allows for visualization of unsupported organisms or integration of multiple strains or closely related species. Multiple association examples of different organisms are provided, including human-mouse phenolog associations. They can be uploaded directly as inputs into POMO. Plots and subsequent filtered findings can be exported as TSV and SVG image files. POMO is free for non-commercial and non-profit usage.

**2. Browser Recommendations**

It is recommended using Firefox or Chrome with POMO as extensive testing has been done on those two browsers. Safari and IE 11+ should work okay but older IE versions do not support secured in-browser reading of local files and other HTML5 features. An alert warning is displayed when such browser versions are detected.

**3. Interface, Views and Layout**

| 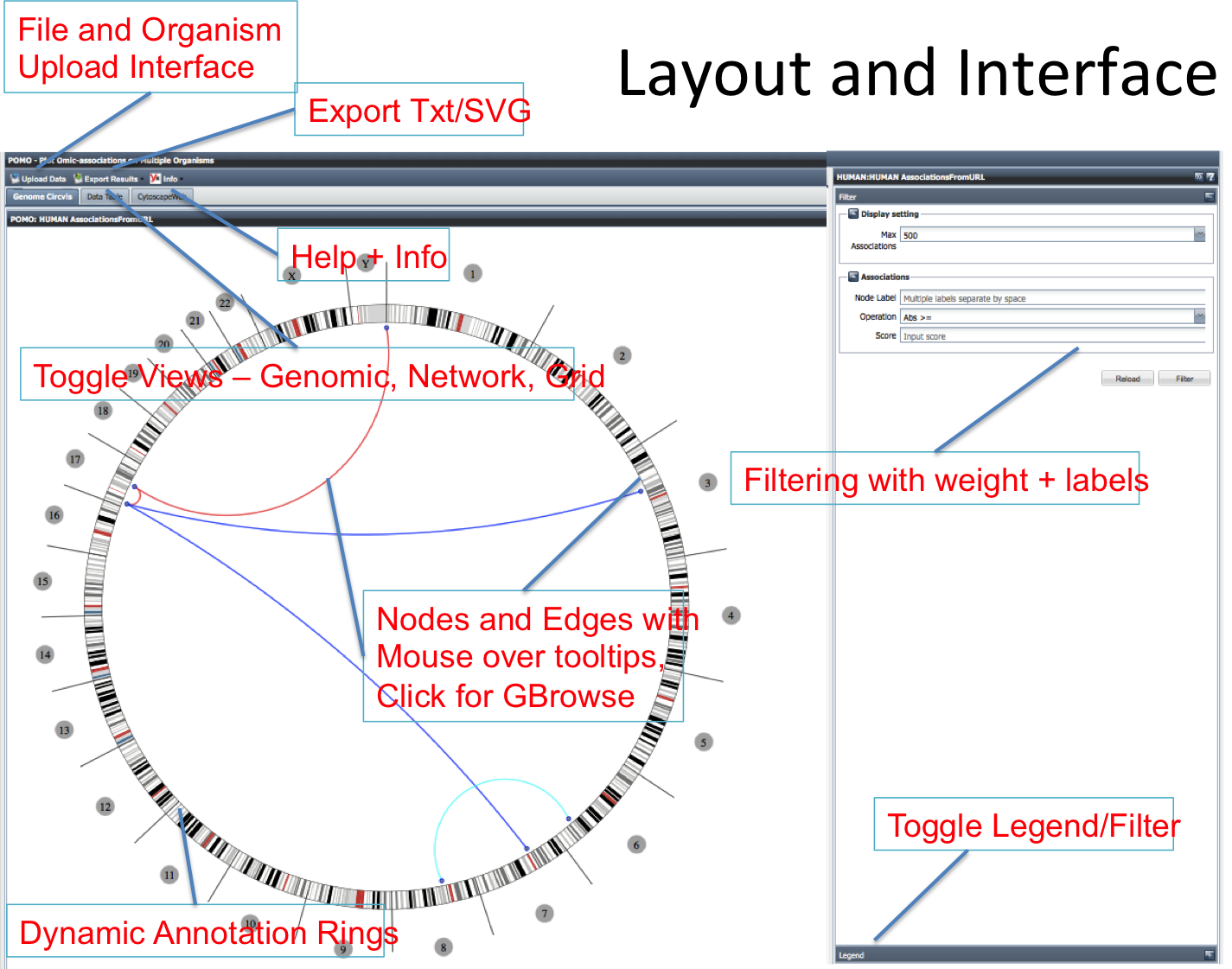 |
| --- |
| *Figure S1. Default view showing example network – all edges, nodes, tiles are clickable and tooltips are shown on mouse over. A color legend, located to the upper right, explains the color encodings to the nodes and edges. An red edge represents ‘Novel’ whereas a black edge is ‘Known’ to selected references.* |

View Components

1. CircVis – Genomic context – chromosomes are drawn as segments of the circumference; their lengths are normalized dependent on their size in nucleotide base counts. A translation service will resolve node labels into chromosome positions that are then drawn as dots onto the perimeter. An association is represented as an arc line between two nodes. Node background and arc colors are used to further annotate different data types and edge weights.

- Outer Rings
  - Cytoband
    - Human and mouse organism selections will display cytoband ring info from UCSC.
    - Custom option will only display applicable associated chromosomes. The organism value will be part of the chromosome label..
  - Annotations
    - The annotation track is intent on giving emphasis on regions within the genome. There can be multiple concentric rings by applying the ‘Append’ function. Heatmaps and histograms are supported. For instance, a user can upload a file using the following format to highlight genomic regions (see Figure S2), see Upload Annotations in the Data upload section for more details:
      - I:68570:96576 green
      - I:200000:300000 green
      - V: 68526: 176532 red
    - Draws green tiles on chrI and a red tile on Yeast chrV

| 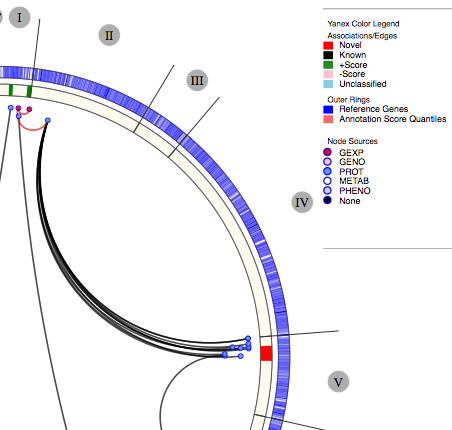 |
| --- |
| *Figure S2. Showing custom annotations. The legend on the upper right corner relates the color representation of edges and nodes. For instance, this graph contains GEXP and PROT nodes by chrI and novel edge between chrI and chrII*. |

- - - Heatmap [example](https://yanex.googlecode.com/files/mouse_heatmap_demo.tsv) - mouse
- **Omics Associations and Nodes**
  - An omics association consists of two nodes that can be mapped to the selected organisms’ chromosomes. The node labels can be gene name, ENSEMBL/ENTREZ or appropriate ID or an actual chromosome position. Explicit chromosome positions add flexibility to node labels since most regions within the genome do not encode for protein-coding genes. The nodes can be annotated with a source type, such as Gene Expression (GEXP). Users can define as many different source types as they wished, but the system currently supports the following type:color mapping (unsupported types are assigned grey node background) - "GEXP":"red","CNVR":"cyan","GENO":"pink","PROT":"cornflowerblue","METAB":"yellow", "MIRNA":"orange", "SNP":"black"
  - An edge can have a weight or an explicit color. Below are some possible human associations, these examples only intent to illustrate the syntax of POMO. Table S1 further describes the node and edge syntax:
  - TP53 BRCA1:GEXP blue
  - TP53 1:100:10000 red
  - TP53 ENSG00000238505:PROT red
  - FYI, the ENSG00000238505 id corresponds to the SNORD11B gene, resolving to chr 2:203156055-203156144

| Node Syntax | Node Example | Edge Syntax | Edge Example |
| --- | --- | --- | --- |
| Gene Label or ID | *TP53* | Label Label weight | *TP53* *BRCA1* -.8 |
| Chr Loci (chr:start:end) | 1:100:10000 | Label:Type Loci color | *TP53*:GEXP 1:100:10000 red |
| Gene/Loci:Type | *TP53*:GEXP | ID Label | *ENSG00000141510* *BRCA1* |
| Unmapped Phenotype | DISEASE:PHENO | Label Phenotype | *TP53* CANCER:PHENO |

*Table S1: Edge and Node Syntax. POMO association nodes are labelled with ENSEMBL, Entrez ids or gene name or chromosome position. Phenotype associations, where one node does not have a genomic location, are supported and useful to describe clinical data.*

2. Cytoscape Web – Network view

Clicking on the CytoscapeWeb tab will display your data in a context free network view.

| 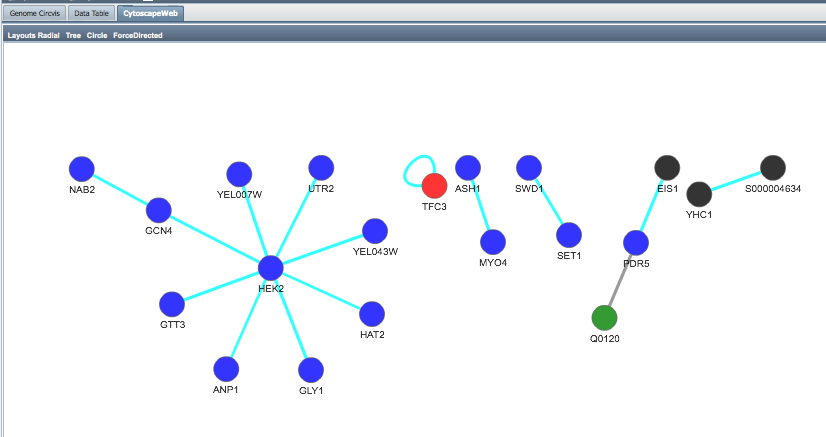 |
| --- |
| *Figure S3. Network view – Force Directed, Tree, Circular and Radial layouts are offered in this view. Nodes can be moved around and clicking on them opens up PubMed keyword search in the organism context.* |

3. Data Table

- The data table view allows users to see the filtered results in the original tabular form. Every column is sortable, ascend and descend. Data will be exported as tab-delimited (tsv) format. These exported files can be used as future POMO inputs.

| 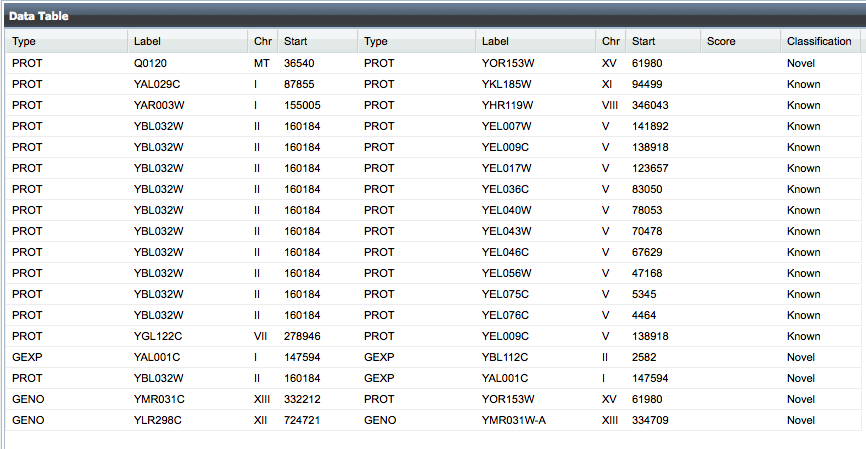 |
| --- |
| *Figure S4. Table sorted by Node A Gene Label.* |

**4. Data Upload and Export**

| 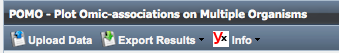 |
| --- |
| *Figure S5. Menu Options* |

**Uploading Data**

- The core functionality of POMO is the ability to upload omics association text files and immediate plotting of the association graph in multiple views. The supported files are txt (space separated), tsv (tab), csv and Simple Interaction Format (sif) files. Cytoscape exported omics SIF files and POMO exported files, including filtered subsets, can be used as inputs.
- Here are some [examples](https://code.google.com/p/pomo/wiki/ExampleInputs). The table below outlines the organisms supported:

| Organism | Species/Build | Source | URL |
| --- | --- | --- | --- |
| Human | H. Sapiens  (GRCh37.p11) | ENSEMBLE | <http://www.ensembl.org/Homo_sapiens/Info/Index> |
| Fly | D. melanogaster  (BDGP5) | Fly base | <http://flybase.org/> |
| Mouse | M. musculus  (GRCm38.p1) | MGI | <http://www.informatics.jax.org/> |
| Worm | C. elegans  (WBcel235) | Worm base | <http://wormbase.org/> |
| Yeast | S. cerevisiae  (EF4) | SGD | <http://www.yeastgenome.org/> |
| Zebra fish | D. rerio  (Zv9) | ZFIN | <http://www.zfin.org/> |
| Arabidopsis | A. thaliana  (TAIR10) | TAIR | <http://www.arabidopsis.org/> |
| Rice | O. sativa  (MSU6) | MSU | <http://rice.plantbiology.msu.edu/> |
| Tomato | S. lycopersicum (SL2.40) | SolGenomics | <http://solgenomics.net/> |
| E. Coli | K-12  (MG1655) | ecocyc | <http://ecocyc.org/> |

*Table S2. Supported Organism References. POMO has build in reference support for human and and a broad range of model organisms. Other organisms and builds will be added as demanded. Node labels can be gene name, chromosome position or ENTREZ/ENSEMBL id.*

- It is important to note that your data is not being shared or stored with any back end servers. We felt it was important to reduce the barrier of dependency on databases and also give the flexibility of sharing data to the user. Supported data formats are:
  - txt/tsv/csv/sif
  - txt/tsv/csv/sif are text files that can be opened in any text editor and tsv/csv can be viewed as spreadsheets in Excel. It is important to export them as tsv/csv (tab separated values/comma separated values) for POMO uploads.
- Nodes are label with ENSEMBL or ENTREZ gene id, gene name or chromosome positions. Unmapped associations/edges are defined when one of the nodes are mappable while the other node is annotated with PHENO source type. A tile will be plotted in the outer most chr 17 q22 area for edge below.
  - CANCER:PHENO BRCA1
- Nodes have the following format:

<ENSEMBLID/gene name>(:<GENO|GEXP|PROT|…>)

- Nodes can also be given in chromosome position format
  - Chr:start:stop:source
    - 5:196948:198519:[GEXP]
- The text file has to be defined in this way:

nodeA[:optional source]\tnodeB[:optional source]\t[optional score/color]

- - YAL029C:PROT\t YER021W:GEXP \t .0001
- SIF [Example](http://yanex.googlecode.com/files/pheno_example.sif)
  - YER021W PP YMR102C YBL032W YEL036C
    - This means that YER021W is associated with YMR102C YBL032W YEL036C , which are consider Protein-Protein interactions.
- Some common interaction types used in the Systems Biology community are as follows and they are supported in POMO:

pp .................. protein – protein interaction

pd .................. protein -> DNA

pr .................. protein -> reaction

rc .................. reaction -> compound

cr .................. compound -> reaction

gl .................. genetic lethal relationship

pm .................. protein-metabolite interaction

mp .................. metabolite-protein interaction

- - GROWTH_MED2% PHENO_GEXP YMR102C YLR298C YMR031W X:10000:20000
    - It is also possible to define PHENO non-genomic nodes such as GROWTH_MED2% and associate them to genomic features, though these associations will only be visible in the CytoscapeWeb view

| 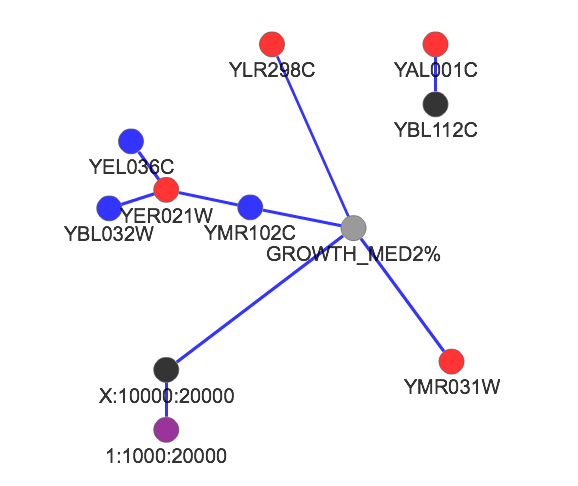 |
| --- |
| *Figure S6. SIF graph – node background and edge color match RE color legends.* |

- For filtering purposes, the optional score needs to be a numeric value

| 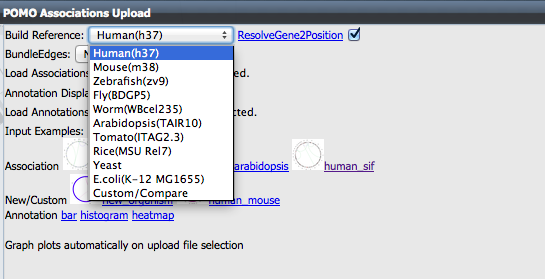 |
| --- |
| *Figure S7. Upon clicking on ‘Browse’ – select appropriate network file and the plotting will be automatic. The speed of uploading and plotting are highly dependent on file and network speed. An initial default maximum limit of 500 edges is defined; though this setting can be updated on the filtering panel using the pulldown, up to 20,000 edges are supported. Testing on a 2013 MacBook Air with 8 GB RAM, it takes 1 second to upload/plot 2000 yeast protein-protein edges. It is our experience that a network becomes a hairball around the 1000 edges, the filtering dynamically updates the graph.* |

- Edge bundling – Genome wide association/network plots can become very dense and 1000 edges or more result in hairballs. As discussed earlier, filtering by scores or gene memberships helps. Another possible solution is to bundle edges. POMO implemented a bundling method using nearest neighbor with a weight threshold option. Starting nodes of edges within a user defined range are grouped together; for clarity, these bundled edges are color gray. This option applies for URL POMO invocations. See Figure S8 for details.

| 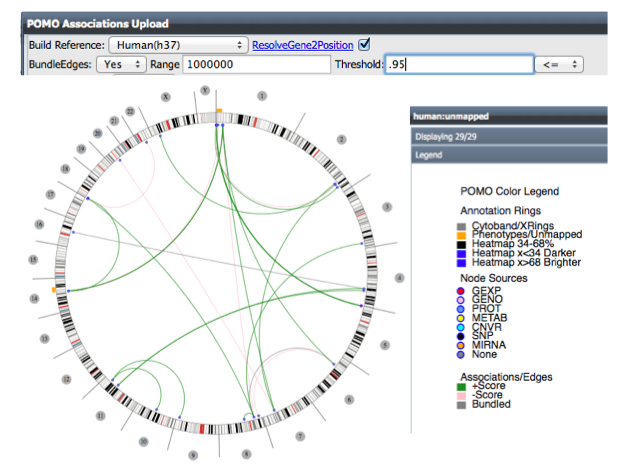 |
| --- |
| *Figure S8. Edges with nodes in the same chromosome and starting nodes within range of defined nucleotide bases, in this case, 1000000, are bundled and plotted as gray edges.* |

**Associations on cloud and URL address**

POMO works with associations already hosted on the cloud, this implies that this file’s security has been set as public accessible. For testing or small association sets, the associations can be directly defined in POMO addresses (first example). The second URL defines associations defined in a file called human_labels.sif that is stored on google code repository that is HTTP accessible

- <http://pomo.cs.tut.fi/?associations=1:1000:10000:,BRCA1,red;tp53,BRCA1,red;TP53,FOXP1,blue;TP53,egfr,blue;mos,myb&organism=human>
- <http://pomo.cs.tut.fi/?fileurl=https://yanex.googlecode.com/files/human_labels.sif&organism=human>

Unresolved Associations

There will be scenarios where association node labels will not be able to resolve to the selected reference, whether it is because of typos or unsupported reference versions. The system keeps track of all unresolved associations and an info dialog box is shown, like the following:

| 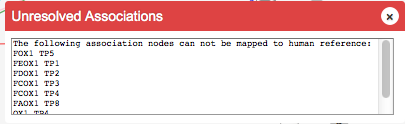 |
| --- |
| *Figure S9. Upon detecting associations where both nodes are not resolvable to reference; users are informed with a dialog box.* |

Bundle edges with URL

Using URL parameter mapping, the bundle algorithm applies, below is an example where bundled is set to ‘yes’, bfunction to ‘greater’ in application of threshold of ‘.90’, meaning that all edges with score > .90 are excluded from bundling.

- http://pomo.cs.tut.fi/?fileurl=https://yanex.googlecode.com/files/unmapped.tsv&organism=human&bundled=yes&bfunction=greater&bthreshold=.90

**Upload Annotations**

- Users may upload custom annotation tracks to highlight or give special meaning to certain genomic regions. For instance, copy number metrics is represented by the assigned color to the chromosome region. The data format is simple: Location and Gene Color or value. Figure S8 below depicts four annotation rings: the standard human cytoband, bar, heatmap, and histogram. Bar is the default type. To define a heatmap or histogram, the annotation file’s first line needs to define the type and then rows following will define coordinate/gene with color/value. Here is an example for heatmap and histogram, focusing on chr 17 and its proximal neighbors of the provided figure:
  - #heatmap blue:red 0:1
  - 17:400000:8000000 .3
  - 17:8000000:18000000 .7
  - 17:28000000:48000000 .02
  - 17:48000000:68000000 .9
  - 17:68000000:98000000 .99
  - #histogram 0.02:4.8
  - 16:84756864:908793700 red:4.6
  - 17:50000000:60000000 blue:2.6
  - 18:1:108793700 red:4.1
- Notice that for heatmap, it can be one or two colors, and then min:max

| 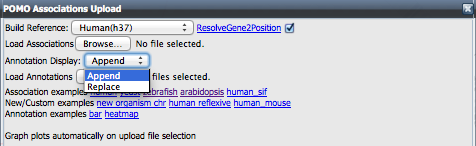 |
| --- |
| 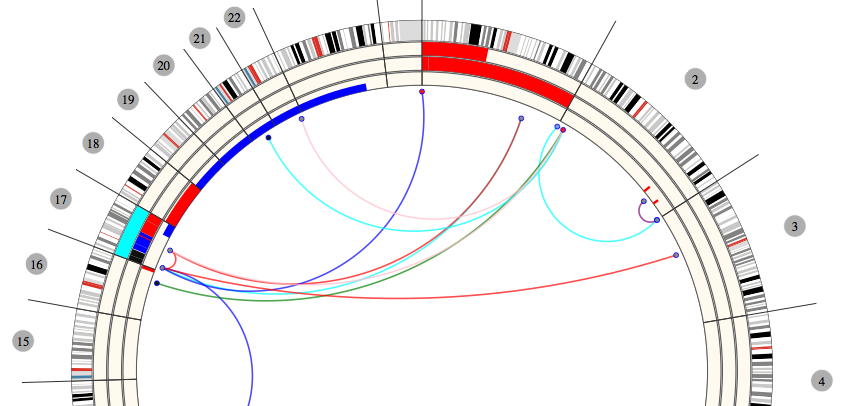   \| *Figure S10. Chromosome annotation bar plots can be dynamically appended. Bar, heatmap and histograms depictions are supported, with the height or variation of the color intensity representing the value in relation to the relative min max.* \| \| --- \| |

Export:

| 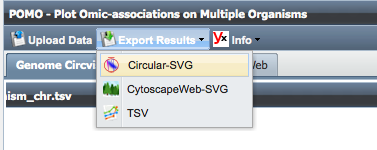 |
| --- |
| *Figure S11. Export functions. The user has the option of exporting TSV text and SVG image files for Circular and CytoscapeWeb views at every iteration of the dataset. SVG files can be converted to high quality png or tiff files using free tools such as Inkscape.* |

- SVG - A new browser window/tab will be opened with full size views and the image files can be downloaded with File -> Save Page from browser menu.
- Exported results are prepend with the filename uploaded – the TSV (filtered) results can be directly used as future POMO inputs.

**5. Filtering**

The example dataset applied here is a set of high quality yeast protein-protein interactions (von Mering et al. 2002 and Lee et al, 2002) released as part of Cytoscape, available here.

<https://docs.google.com/file/d/0B7Y4_iaYnqExWTAxMkdDLWZ5U0E/edit?usp=sharing>

First, yeast is selected as the current organism. Then, the association file is uploaded and the graph is drawn using the default limit of 2000 rows. Figure S11 below is the resultant plot. Often, networks with thousands of edges formed dense graphs that are not informative; the following section will go over usage of filtering in exploration of such graphs.

| 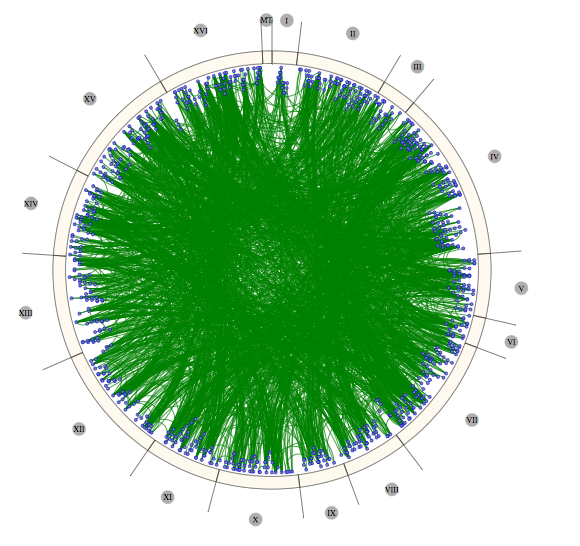 |
| --- |
| *Figure S12. 6888 High quality protein-protein interactions are uploaded into a Firefox web browser and 2000 edges are plotted. Working on a MacBook Air laptop, the uploading, resolving and drawing took ~2 seconds*. |

**Parameter/Filtering** Panel

| 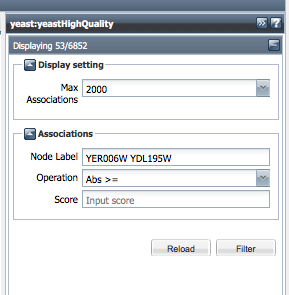 |  |
| --- | --- |

*Figure S13. Filtering options – Clicking on the Filter button will bring back all the edges, or up to selected max count (order by position in file) that meet the filtering parameters – in this case edge between the two nodes labeled YER006W and YDL195W, there is no weight associated with this dataset, otherwise it is possible to combine the node label check with edge weight. Figure S13 contains the 53 resultant edges displayed in circular, tabular and network views.*

The filtering and re-plotting usually is instantaneous for datasets up to 10000 rows with display limit of 1000, and about 2 seconds for display limit of 2000. Clicking on the Reload button will re-plot the unfiltered associations.

| 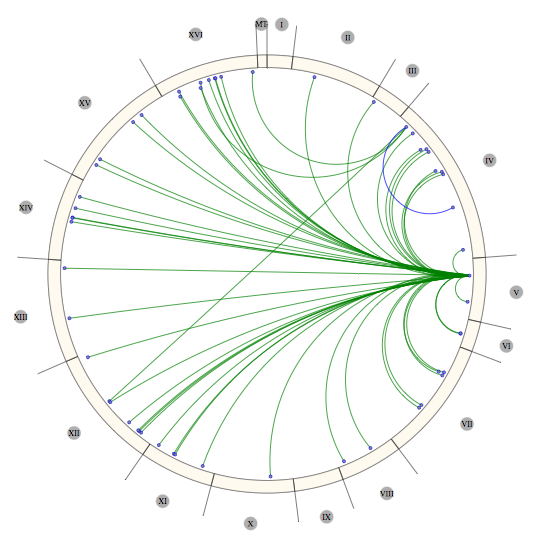 |
| --- |
| 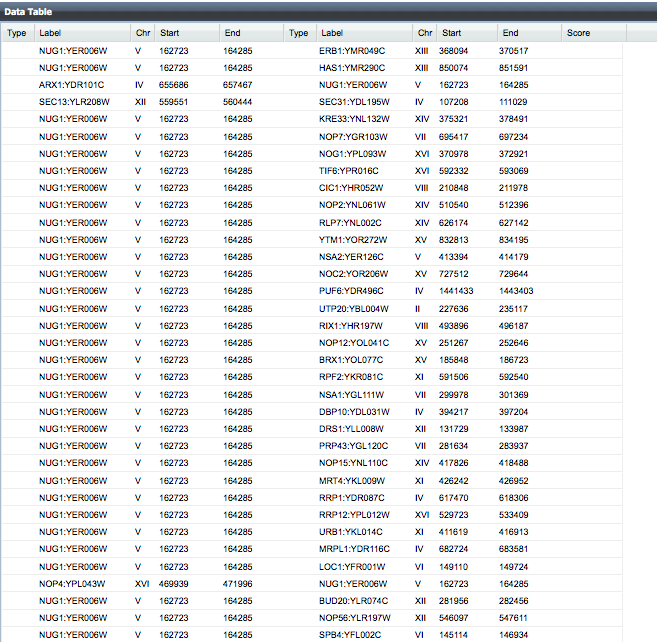 |
| 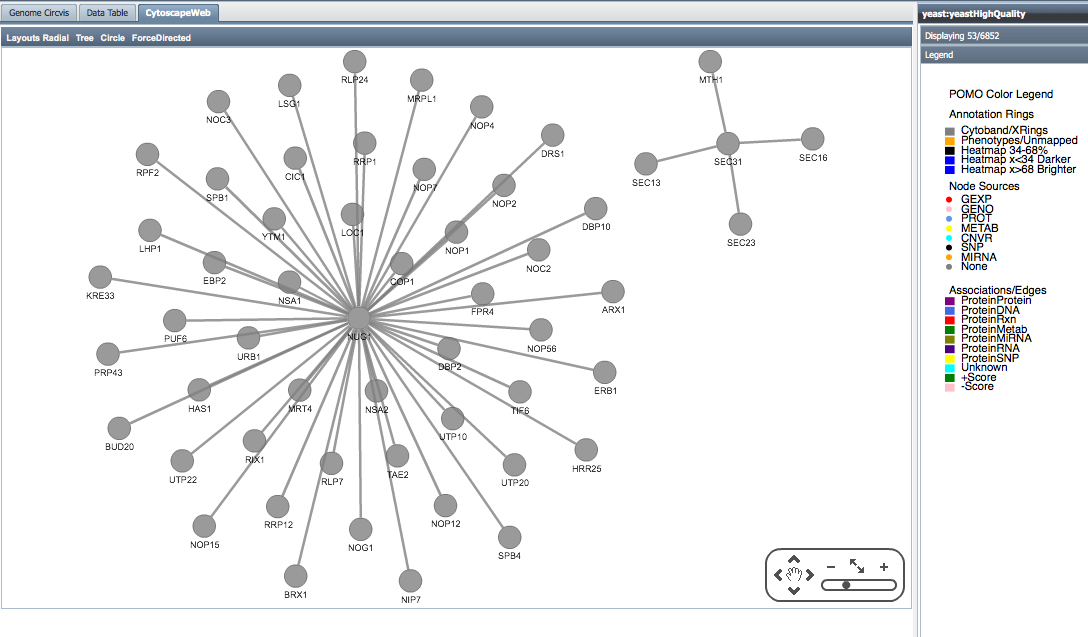   \| *Figure S14. It is often helpful to use the ‘Data Table’ for validation as the gene labels are shown with their chromosome positions. The Cytoscape Web network view provides several layouts and aids in detection of disjoint sub-networks.* \| \| --- \| |

1. Custom Organism/Homology – A novel feature we developed for POMO is the ability to plot associations for custom or unsupported organisms and to integrate pairwise species and strains comparisons. By defining the new (NEW checkbox) chromosome names and their lengths in nucleotides, POMO acts as a canvas, and the input chromosomes become the de facto references. The association node labels need to be chromosome position based and its chromosome name need to be part of the input provided. The example below provides more details and the scenario’s data sets are included in the code repository.

| **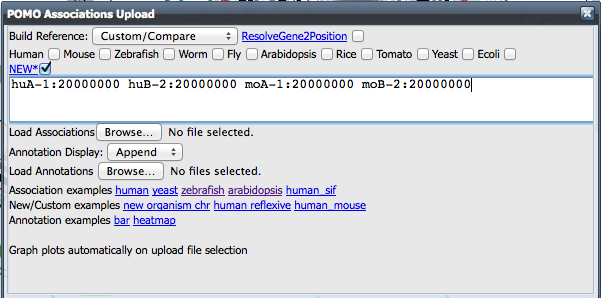** |
| --- |
| **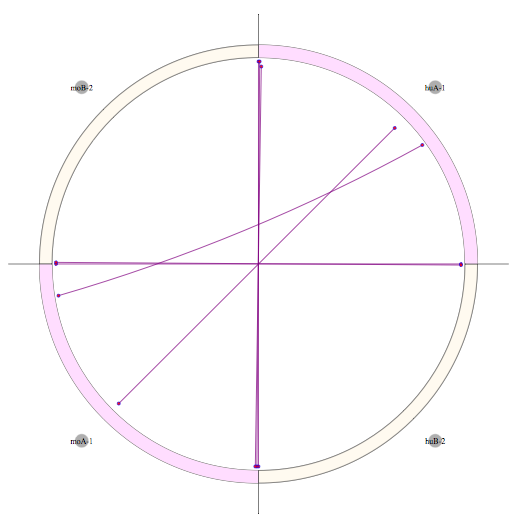**   \| *Figure S15. After entering in new chromosome info as shown, this* [*example association file*](https://yanex.googlecode.com/files/new_organism_chr.tsv) *is uploaded and the edges plotted.* \| \| --- \| |

1. Upload Data -> Custom/Compare is selected for Organism

2. NEW is checked

3. Enter following in chromosomes field (see Figure S14):

huA-1:20000000 huB-2:20000000 moA-1:20000000 moB-2:20000000

4. The example edges are (tab separated):

huA-1:1000:2000:GEXP moA-1:2000:2001:GEXP purple

huA-1:71000:90000:GEXP moA-1:102000:120001:GEXP purple

huA-1:181000:190000:GEXP moA-1:200000:300001:GEXP purple

huA-1:10010000:10002000:GEXP moA-1:10010000:10010008:GEXP purple

huA-1:12001000:13002000:GEXP moA-1:18002000:20000201:GEXP purple

huB-2:1000:2000:GEXP moB-2:2000:2001:GEXP purple

huB-2:71000:90000:GEXP moB-2:102000:120001:GEXP purple

In the last example we will go over is the plotting of associations between two related organisms. The scenario here covers human-mouse homologies, the associations is a subset taken from <http://www.informatics.jax.org/homology.shtml>

The following associations are stored as a tsv file and then uploaded:

human:TP53:GEXP mouse:TP53:GEXP blue

human:1:9500000:10000000:PROT mouse:1:7000:7005:PROT red

human:FOXP1:GEXP mouse:BRCA1:GEXP purple

human:FOXP1:GEXP mouse:FOXP3:GEXP purple

human:BRCA1:GEXP mouse:TP53:GEXP blue

human:BRCA1:GEXP mouse:BRCA2:GEXP blue

human:BRCA2:GEXP mouse:BRCA1:GEXP blue

human:PON2:GEXP mouse:PON2:GEXP blue

human:PCSK9:PROT mouse:PCSK9:PROT red

| **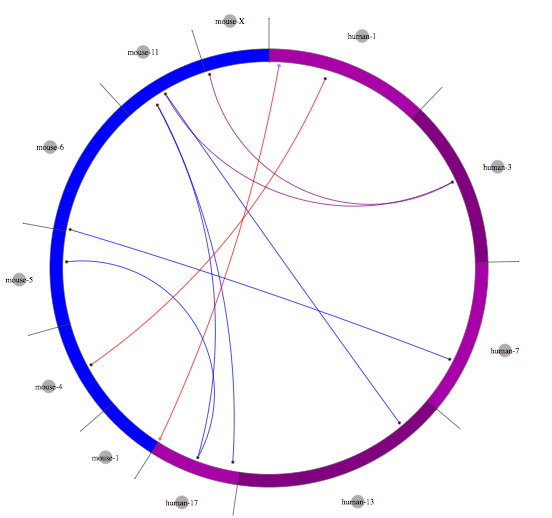** |
| --- |
| *Figure S16. The associations represent human to mouse homologies and the chromosome circular arcs labeled and colored dependent on the organism hosts.* |

**In addition, we recommend looking at** [**http://www.phenologs.org**](http://www.phenologs.org) **for more in depth information on orthologs between organisms.**

Custom/New Organism options:

1. Select Two Organisms
   1. Select One Organism
      1. Reflexive (Implying that the organism reference is mirrored, for example, if yeast is selected, then the circular arcs are divided and represent two yeast genomes)
2. Compress2GeneView
3. NEW
   1. Enter Organism:chromosome:bases
      1. huA-1:100000 moA-1:100000 means that all association nodes must mapped and be in range of huA-1 and moA-1

7. Architecture and Libraries – POMO is open sourced and implemented using modern web technologies. The source code, issue tracker and additional documentation are hosted at: http://pomo.googlecode.com

| **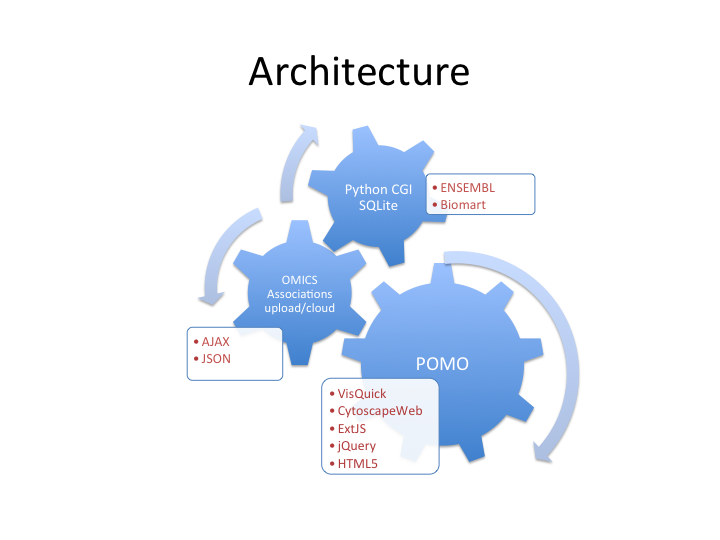** |
| --- |
| *Figure S17. Using modern web technologies omics associations can be directly uploaded or referenced as a parameter if stored on cloud. Lightweight SQLite instances store organism references and annotations from Ensembl and other public resources.* |

Library components:

- VisQuick - https://code.google.com/p/visquick/
- CytoscapeWeb - http://cytoscapeweb.cytoscape.org/
- jQuery - http://jquery.com/
- ExtJS - http://www.sencha.org/
- Messi - http://marcosesperon.es/apps/messi/
- Python - http://www.python.org/
- SQLite - http://www.sqlite.org/
